# Gastrointestinal Manifestations of Sarcoidosis: A State-of-the-Art, Comprehensive Review of the Literature—Practical Clinical Insights and Many Unmet Needs on Diagnosis and Treatment

**DOI:** 10.3390/ph17091106

**Published:** 2024-08-23

**Authors:** Salvatore Nicolosi, Maria Chernovsky, Darina Angoni, Michael Hughes, Giulia Bandini, Zsuzsanna McMahan, Marta Maggisano, Francesco Salton, Lucrezia Mondini, Mariangela Barbieri, Gianluca Screm, Marco Confalonieri, Elisa Baratella, Paola Confalonieri, Barbara Ruaro

**Affiliations:** 1 Pulmonology Unit, Department of Medical Surgical and Health Sciences, University of Trieste, 34149 Trieste, Italyangoni.darina@gmail.com (D.A.); marta.maggisano@gmail.com (M.M.);; 2 Division of Musculoskeletal and Dermatological Sciences, Faculty of Biology, Medicine and Health, The University of Manchester & Salford Royal NHS Foundation Trust, Manchester M6 8HD, UK; 3 Department of Experimental and Clinical Medicine, Division of Internal Medicine, Azienda Ospedaliero Universitaria Careggi, University of Florence, 50134 Florence, Italy; giulia.bandini@unifi.it; 4 McGovern Medical School, The University of Texas Health Science Center at Houston, Houston, TX 77030, USA; 5 Radiology Unit, Department of Medical Surgical and Health Sciences, University Hospital of Cattinara, 34149 Trieste, Italy

**Keywords:** gastric sarcoidosis, hepatic sarcoidosis, spleen sarcoidosis, colon sarcoidosis, gastrointestinal sarcoidosis treatment, GERD

## Abstract

This comprehensive literature review explores the involvement of the gastrointestinal (GI) tract in sarcoidosis, a multisystem granulomatous disorder of unknown etiology. GI sarcoidosis presents a diagnostic and therapeutic challenge due to its rarity and nonspecific clinical manifestations, including overlap with other gastrointestinal diseases. We conducted a comprehensive screening of articles addressing the clinical features, diagnostic approaches, and treatment strategies for GI sarcoidosis. Our findings reveal that GI sarcoidosis can affect any part of the gastrointestinal tract, with the stomach and small intestine being the most involved. Clinical presentations range from asymptomatic cases to severe complications such as obstruction and perforation, with reflux being a common symptom. Diagnosis is often delayed due to the nonspecific nature of symptoms and the need for histopathological confirmation. Therapeutic approaches are poorly defined, typically involving corticosteroids as the mainstay of treatment. However, the long-term efficacy and safety of these treatments remain uncertain in this patient group, given the significant risks and complications associated with prolonged glucocorticoid therapy. There is a clear need to develop accurate diagnostic protocols to distinguish GI sarcoidosis from other conditions and to establish standardized therapeutic guidelines to optimize patient outcomes. Further research is essential to enhance our understanding and management of this complex condition.

## 1. Introduction

Sarcoidosis is a rare, multisystemic granulomatous disease characterized by non-caseating granulomas, most commonly in the lungs and intrathoracic lymph nodes. Gastrointestinal involvement is particularly rare and can occur as part of systemic disease or as an isolated condition. In the literature, articles describing isolated gastric sarcoidosis are very limited, highlighting not only the rarity of the condition but also the challenges in achieving an accurate diagnosis. This scarcity of cases underscores the importance of considering it in differential diagnoses despite its infrequency [1,2,3].

The histopathological hallmark of sarcoidosis, whether it affects the lungs or other organs, is the presence of non-necrotizing, or non-caseating, granulomas, consisting of a tightly packed center surrounded by a looser outer layer. Finally, the whole structure is surrounded by lamellar rings of hyaline collagen with scarce cellularity [1]. Sarcoidosis is a multi-organ disease characterized by the formation of non-caseating granulomas, which are clusters of immune cells that form in response to an unknown trigger. While the exact cause of sarcoidosis remains unclear, it is believed that various factors, such as viruses, pollutants, or allergens, could act as irritant triggers. In most cases, however, the specific triggering substance is not identified. The granuloma formation may involve immune responses to potential infective agents, although this has not been definitively established. The irritant, which is then phagocytized and broken down by dendritic cells and macrophages, is presented to the cluster differentiation (CD)4+ T helper cells, which secrete pro-inflammatory interleukins and recruit macrophages, creating the tightly packed center of the granuloma. Subsequently, B lymphocytes and T lymphocytes aggregate around the center, creating the second, looser layer of the granuloma. Finally, fibroblasts settle on the outside of the granuloma and secrete collagen, creating the outer lamellar rings and forming a fibrous matrix [1,3]. Immunohistochemical markers can help in the diagnosis of sarcoidosis by identifying specific immune cells within granulomas, such as CD68 and CD163 for macrophages and the CD4/CD8 ratio for T lymphocytes. These markers also help exclude other granulomatous diseases like tuberculosis and fungal infections. However, they are not specific to sarcoidosis and should be interpreted in conjunction with clinical and radiological assessments [4].

Diagnosis of sarcoidosis is often difficult, as symptoms widely vary depending on the organs involved and the severity of the disease. In 2020, the American Thoracic Society (ATS) released its first-ever statement regarding the diagnosis of pulmonary sarcoidosis, basing it on three criteria: (1) a compatible clinical and radiological manifestation, (2) histological evidence of non-caseating granulomas and (3) the exclusion of any alternative cause [5]. The thoracic radiological manifestation is based on the Scadding scale, which divides sarcoidosis into four classes according to thoracic radiological presentation. These classes are not related to disease severity and progression and only indicate organ involvement within the thorax; although originally used with simple chest radiography, it can be adapted to the more commonly used computerized tomography scan (CT scan) [6].

These guidelines, however, are only viable for diagnosing pulmonary or mediastinal sarcoidosis, making diagnosing any extrathoracic manifestations of the disease less uniform and much more dependent on the individual physician’s experience examining the patient. Consequently, a tool for assessing organ involvement in sarcoidosis, named ACCESS, or A Case Control Etiologic Study of Sarcoidosis, was first suggested in 1999 [6]. Based first and foremost on bioptic findings in 736 enrolled patients, the tool defines the probability of multi-organ involvement in patients with a single tissue finding of sarcoidosis via biopsy. It covers most to least frequently involved organs and systems and relies both on the physician’s pursuit of an accurate diagnosis and routine laboratory and instrumental follow-up such as radiographic imaging, respiratory function tests (RFTs), hemochrome and blood serum analysis, and other organ-specific tests to help determine involvement rate. Results suggested that organ involvement can be classified into three main groups: (1) likely involved (e.g., lungs, skin, liver, eyes, or calcium metabolism), (2) unusual but clinically significant (nervous system, heart, and kidneys), (3) and other sites (e.g., spleen, bones, bone marrow, muscles, upper airways, ears, salivary glands, and extrathoracic lymph nodes) [6,7].

This approach, however, only offered a preliminary assessment and did not allow for a more minute characterization of the condition the involved organ or system were in. In 2007, a method called STAI (sarcoidosis three-dimensional assessment instrument) was introduced for a more accurate and revised vision of the specific organ. The method was based on three axes: organ involvement, disease severity, and disease activity [8].

The first axis in the STAI method expands upon the findings of ACCESS, adding another category dubbed “other organs”, which were not included previously, as well as accounting for alternative diagnostic methods outside of biopsies, such as liver involvement being defined by a threefold elevation of hepatic enzymes, compatible Computerised Tomography (CT) scan, and elevated alkaline phosphatase [8]. The second axis allows for assessing the severity of sarcoidosis, categorizing results into one of four classes I-IV with growing severity of limitation to function. The third axis assesses the disease activity as part of an effort to minimize unnecessary steroid treatment [8].

The first axis specifically offers an important update missing from the ACCESS approach. As mentioned, sarcoidosis is a systemic condition with visible effects beyond just the fifteen organs originally examined. The GI system outside of the liver and spleen is rarely involved, and unfortunately, the STAI system does not offer any benefits for their differential diagnosis over the ACCESS one. Such cases of GI sarcoidosis that do not involve the liver or spleen have been documented in the past mostly as sparse case reports and never explicitly cataloged in a single comprehensive review of the literature [9]. Moreover, the STAI method has not yet been validated for sarcoidosis assessment and diagnosis.

## 2. Material and Methods

**Figure 1:**
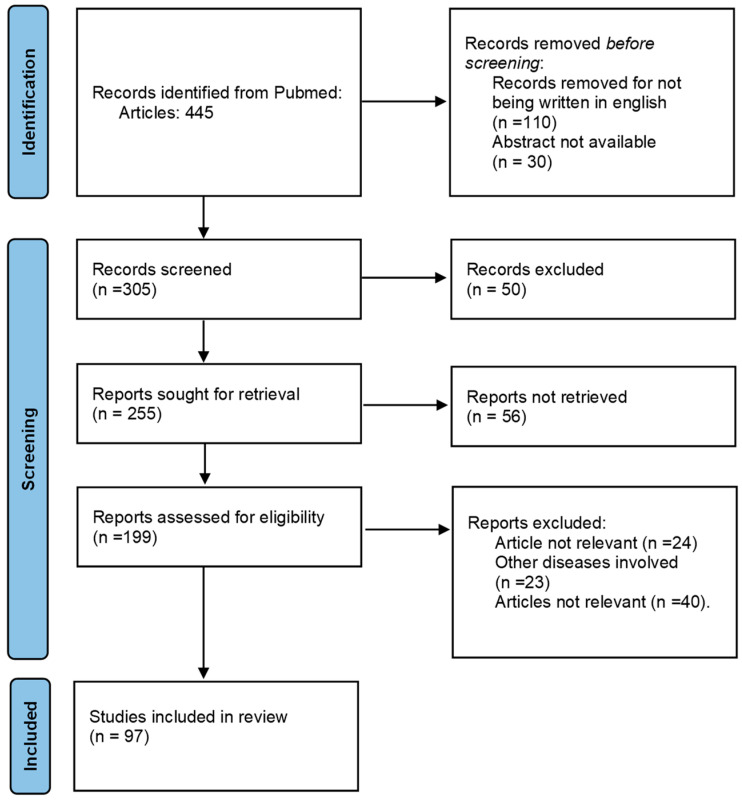
Flow chart of the diagnosis and treatment of sarcoidosis involving the gastrointestinal tract articles.

A comprehensive literature review was conducted to investigate sarcoidosis involvement of the whole GI tract. Articles included in PubMed and Google Scholar were searched using the keywords “gastric sarcoidosis”, “digestive tract sarcoidosis”, “stomach sarcoidosis”, and “gastric sarcoid granulomas”. For liver, spleen, and pancreatic sarcoidosis, PubMed was searched for articles published from January 1967 to May 2024 using the terms “hepatic sarcoidosis”, “hepatic sarcoidosis involvement”, “hepatic sarcoidosis treatment”, “pancreatic sarcoidosis”, “pancreatic sarcoidosis treatment”, “spleen sarcoidosis”, and “spleen sarcoidosis treatment”, excluding non-English and irrelevant articles. For bowel involvement, PubMed was searched from January 2000 to May 2024 using “colon sarcoidosis”, “large intestine sarcoidosis”, “colon sarcoidosis involvement”, “large intestine sarcoidosis involvement”, “colon sarcoidosis treatment”, “gastrointestinal sarcoidosis”, “gastrointestinal tract”, and “large intestine sarcoidosis treatment”, excluding non-English articles. Inclusion criteria were publications from 1950 to 2024, adult human subjects, and English language, while abstracts without full-text articles and conference abstracts were excluded. Our research yielded 445 articles, with 97 deemed to be most relevant for our review article (Figure 1).

## 3. Sarcoidosis Involvement of the Gastrointestinal Tract

This comprehensive literature review explores gastrointestinal (GI) involvement in sarcoidosis, a multisystem granulomatous disorder. Despite its rarity, GI sarcoidosis presents significant diagnostic and therapeutic challenges due to nonspecific symptoms and overlap with other GI diseases. The GI system is among the rarest, if not the rarest, to be affected by sarcoidosis [10,11]. Manifestations of GI sarcoidosis are heterogeneous, though many manifest in the form of pain and do not affect the GI tract uniformly. Nearly 80% of cases affect the upper GI tract (oropharynx, esophagus, stomach, and duodenum) [11]. Oral cavity sarcoidosis often manifests in swollen gingiva, submucosal nodules, mucosal lesions, and tooth mobility due to bone erosion [12]. Pharyngeal sarcoidosis is frequently asymptomatic but may manifest with a progressive loss of function, such as dysphonia and dysphagia [13]. Esophageal involvement is characterized by dysphagia but may also manifest symptoms stemming from extrinsic compression of nerves, such as neuropathy [14]. Gastric sarcoidosis often presents itself with vague and subtle symptoms that may coincide with that of duodenal sarcoidosis and are, for the most part, a result of subcontinuous mucosal lesions that reduce nutrient absorption capacity and disrupt the physiologic gastric motility [10,11,12,13,14,15]. Small intestinal sarcoidosis may present as an intestinal obstruction, causing stenosis or blockage of the lumen [16]. Large intestinal sarcoidosis does not often cause intestinal blockage but does, similarly to the duodenal and gastric ones, cause weight loss, bowel pain, diarrhea, and constipation [17]. Finally, rectal sarcoidosis may manifest as a rectal mass in affected patients [17,18].

Sarcoidosis manifestations vary depending on the organ or system involved, but treatment is, first and foremost, that of watchful waiting [19,20,21,22,23,24,25,26,27,28,29]. According to the most recent ATS guidelines, the watchful waiting approach is to be preferred over oral steroid-based therapy in case of asymptomatic patients or patients with mild symptoms that do not affect the quality of life. On the contrary, when symptoms become severe or in cases of disease progression, long-term oral steroid-based treatment is the first choice of treatment, and steroid-sparing agents and biological drugs are the second and third lines of treatment, respectively [7,8,9,10,11,12,13,14,15,16,17,18,20,21,22,23,24,25,26,27,28,29,30,31,32,33,34,35,36,37,38].

### 3.1. Gastric Sarcoidosis Involvement

GI involvement in sarcoidosis is rare, occurring in 0.1% to 3.4% of all cases, with symptomatic GI sarcoidosis being even less common, affecting less than 1% of patients. Macroscopic lesions can develop in any part of the digestive tract, with the stomach being the most frequently involved site, seen in approximately 10% of those with gastrointestinal sarcoidosis [19]. Gastric sarcoidosis, whether isolated or as part of systemic sarcoidosis, represents a challenging diagnosis due to its rarity and nonspecific symptoms (Table 1 and Table A1 in Appendix A) [39,40,41,42,43,44,45,46,47,48,49,50,51]. The various symptoms reflect the disease’s patchy mucosal involvement and subtle nature. Thus, the diagnosis requires a combination of high clinical suspicion, imaging, and histopathological confirmation. The association between gastroesophageal reflux disease (GERD) and sarcoidosis likely stems from the inflammatory nature of sarcoidosis, which can cause esophageal motility disorders due to nerve involvement and granuloma formation [14,52,53,54,55,56,57,58,59,60,61,62,63,64,65,66,67,68,69,70,71,72,73]. Diagnosis is often delayed due to the need for histopathological confirmation. While corticosteroids are the mainstay of treatment, their long-term efficacy and safety relative to their long-term important adverse effects remain uncertain (and presumably may exacerbate GERD symptoms), and steroid-sparing agents (e.g., methotrexate, azathioprine, antimalarials) and ursodeoxycholic acid have shown some success. Effective management of GERD through lifestyle modifications, medications, or surgery is also crucial. This review underscores the need for accurate diagnostic protocols and standardized therapeutic guidelines to optimize patient outcomes, highlighting the importance of further research in this area [14,15,16,17,18,19,20,21,22,74,75,76,77,78,79,80,81,82,83,84,85,86,87,88,89].

Patients with known systemic sarcoidosis, particularly those with extrapulmonary manifestations, are more likely to develop GI involvement. Gastric granulomas have been reported in up to 10% of patients with pulmonary sarcoidosis [6,7,8,9,10,11,12,13,14,15,16,17,18,19,20,21,22,23,90,91,92,93,94,95,96,97,98,99,100,101,102,103,104]. Endoscopy is the primary tool for visualizing gastric mucosal abnormalities and obtaining biopsy samples, avoiding delays in diagnosis and treatment. Biopsy and histopathology remain the gold standard for diagnosis, revealing non-caseating granulomas that contain multinucleate giant cells in the absence of crypt abscesses on histology. However, the patchy distribution of these granulomas can lead to false negatives, especially if superficial biopsies are performed. In association with endoscopy, F-FDG-PET/CT may be helpful in patients with sarcoidosis for determining the intrathoracic and extrathoracic extensity of disease, detecting active disease, and accessing the response to treatment, particularly in atypical, complex, and multisystem forms of sarcoidosis. Gallium-67 scan requires multiple visits for assessment and is less accurate, involving increased radiation exposure (~15 mSv) and necessitating imaging up to 48 h after injection, making it a prolonged procedure [24,25,105,106,107,108,109,110,111,112,113,114].

In developed countries, sarcoidosis is the second most common cause of granulomatous gastritis. The differential diagnosis involves ruling out other granulomatous diseases like Crohn’s disease, Whipple’s disease, infections (e.g., tuberculosis, fungal infections, H. pylori), functional dyspepsia, vasculitis, Langerhans cell histiocytosis and malignancies (lymphomas, hematological malignancies, and gastric adenocarcinoma). Histiocytic markers such as CD68 are usually strongly reactive in sarcoid granulomas. Pan-cytokeratin staining, which is seen in malignancy, should be negative. Staining for infectious agents can aid in this exclusion. Although not always pathognomonic, immunohistochemical markers can be useful. A rare association between sarcoidosis and Crohn’s disease is reported, and additional targeted therapy against tumor necrosis factor-α and integrins is associated with the induction of sarcoidosis when used to treat inflammatory bowel disease [26,27].

*Propionibacterium acnes* has been associated with granuloma formation, with 92% positive staining using the PAB antibody reported in Japanese cases of sarcoidosis. Inomata et al. also confirmed this association in the gastric lesion. In a study of five cases of gastric sarcoidosis, *Helicobacter pylori* infection was associated with 40% of cases [113].

The primary treatment for gastric sarcoidosis, like systemic sarcoidosis, involves the use of corticosteroids to reduce inflammation and granuloma formation (to manage active disease and symptoms). In cases where corticosteroids are not effective or cause significant side effects, alternative immunosuppressive agents like methotrexate or azathioprine may be considered [27]. Proton pump inhibitors (PPIs) or Histamine2 (H2) blockers may be used to manage gastric symptoms, such as acid reflux and dyspepsia. Nutritional support is vital, especially in patients experiencing significant weight loss. The prognosis for isolated gastric sarcoidosis is generally good with appropriate treatment. Longitudinal studies have shown that most asymptomatic patients with gastric sarcoidosis typically do not develop GI symptoms over time or exhibit changes in biopsy patterns. However, GI involvement, though rare, is possible in patients with pulmonary and extrapulmonary sarcoidosis. These patients remain susceptible to disease progression even while on high doses of immunosuppressive therapy and may require more prolonged and intensive management. Therefore, patients with sarcoidosis and persistent gastrointestinal symptoms should be evaluated with endoscopy and biopsy, as described in the work of Hassan et al. [23].

The prognosis for isolated gastric sarcoidosis is generally good with appropriate treatment. Longitudinal studies have shown that most of the asymptomatic patients with gastric sarcoidosis typically do not develop GI symptoms over time, nor do they exhibit changes in biopsy patterns; in contrast, patients with pulmonary and extrapulmonary sarcoidosis remain susceptible to disease progression, even when on high dose immunosuppressive therapy, and may require more prolonged and intensive management [32].

### 3.2. Small Bowel Sarcoidosis Involvement

GI sarcoidosis, particularly in the small bowel, is rare (less than 10 cases reported in the extant literature) but a clinically significant manifestation of sarcoidosis. Patients with small bowel sarcoidosis often present in their fifth or sixth decade of life and usually have multisystem sarcoidosis. Imaging modalities such as computed tomography (CT) are essential for patients presenting with abdominal pain with known sarcoidosis. Endoscopic examination is the preferred initial investigative approach for patients with diarrhea. Diagnosis relies heavily on histopathological confirmation and exclusion of other granulomatous diseases such as tuberculosis, characterized by the absence of necrosis [1], or Whipple’s disease, which may be identified via the periodic acid-Schiff (PAS) coloration [33]. Histological findings may not always be clear-cut, however. Crohn’s disease may present itself with either diffuse nonspecific inflammation, diffuse granulomatous inflammation or focal granulomas, which are present in nearly 50% of patients and are virtually indistinguishable from the sarcoid granuloma [34]. In such cases, differential diagnosis relies on other criteria such as the age of onset or involvement of organs outside the gastrointestinal tract, as well as serological markers such as the presence of anti-neutrophil cytoplasm antibodies (ANCAs) or anti saccharomyces cerevisiae antibodies (ASCAs) [35].

These cases underscore the importance of considering sarcoidosis in the differential diagnoses of patients with sarcoidosis for acute abdominal presentations and the necessity of timely surgical intervention to prevent complications such as perforation or life-threatening hemorrhagic ascites. Treatment is tailored based on symptom severity, with corticosteroids being the cornerstone of therapy [36,37,38,39].

### 3.3. Hepatic Sarcoidosis

Hepatic sarcoidosis is characterized by non-caseating granulomas in the liver, biliary tract, and gallbladder. There are no formalized diagnostic criteria for hepatic sarcoidosis; diagnosis typically involves a clinical history of systemic sarcoidosis and liver biopsy evidence of non-caseating granulomas. The prevalence of hepatic involvement in patients with systemic sarcoidosis varies widely between studies, ranging from 3.6 to 30% [40,41,42,43,44]. However, autopsy studies have demonstrated a higher prevalence of disease (up to 70%), highlighting that hepatic involvement is a frequent, yet often under-recognized, complication of systemic sarcoidosis [45]. While many patients are asymptomatic despite granulomas on biopsy, abnormal liver enzymes, or radiological findings [41], about 15% experience hepatomegaly, right upper quadrant pain, and systemic symptoms (e.g., fatigue, fever, and arthralgias), which are nonspecific but are present in most patients with active liver sarcoidosis [46]. Liver function test abnormalities primarily show a cholestatic pattern [47]. Hepatic sarcoidosis manifesting solely as biliary sarcoidosis with cholestasis symptoms is documented in a few case reports. In these instances, ERCP with brush cytology was necessary when cholangiocarcinoma was highly suspected [44,45,46,47,48,49,50,51,52]. Despite hepatic sarcoidosis being generally a chronic disease with a benign course, a minority of patients may develop chronic liver disease that advances to portal hypertension associated with risks of variceal bleeding, progression to end-stage cirrhosis, and increased incidence of hepatocellular carcinoma (HCC) [43,44,45,46,47,48,49,50,51,52,53,54,55,56,57,58]. Active treatment is not indicated for all patients with hepatic sarcoidosis. Observation is appropriate for those with asymptomatic liver disease, mild elevations in serum liver enzymes, and normal liver function without evidence of cholestasis [59]. An interesting aspect to consider is the connection between sarcoidosis and malignant tumors. As described in the work of Cohen et al. [60], three types of associations exist, the first of which is the association between sarcoidosis and liquid tumors such as lymphomas and bears little relevance to the gastrointestinal system, while the other two pose a direct connection. The second type of association consists of patients with sarcoidosis developing subsequent malignant tumors or vice versa, among which hepatocellular carcinoma is one of the most noted examples [61,62]. The third type of association is not considered a full-blown sarcoidosis in and of itself but rather a sarcoid-like reaction in the case of malignant tumors, in which non-necrotizing granulomas develop and are confined to the regional lymph nodes. Such a reaction, however, lacks the ‘systemic’ aspect of sarcoidosis as it is confined to the regional lymph nodes and thus can only be considered a sarcoid-like reaction [60,61,62,63].

In symptomatic patients, the treatment goal for hepatic sarcoidosis is to control symptoms and prevent progression to cirrhosis, portal hypertension, and liver transplant. However, responses to conventional therapies are variable, and no standardized protocols exist. In our review of the literature, we found three articles (shown in Table 2) that are focused on the treatment of hepatic sarcoidosis outcomes. Corticosteroids, often combined with ursodeoxycholic acid (UDCA) for cholestasis, are the first line of therapy; they reduce hepatic granulomas and alleviate symptoms like fever, fatigue, pruritus, and weight loss, but their long-term benefits are unclear [46,47,48,49,50,51,52,53,54,55,56,57,58,59,60,61,62,63,64]. UDCA helps reduce cholestasis symptoms and potentially delays disease progression, benefiting patients unresponsive to or dependent on steroids [41,42,43,44,45,46,47,48,49,50,51,52,53,54,55,56,57,58,59,60,61,62,63,64,65,66,67,68,69,70,71,72]. When patients require a step up in therapy, azathioprine, methotrexate, cyclophosphamide, and infliximab have shown some benefits, but the extant literature lacks strong evidence. Studies indicate varying effectiveness, with some patients responding with changes in the cholestatic enzyme levels and others not. Sedki et al. [42] identified that antimetabolites elicited a statistically significant change in ALP in individuals with preexisting ALP elevation (Table 2). In contrast, patients receiving oral glucocorticoids or biologic agents showed a decrease in their ALP level, which was not statistically significant, and only two patients received orthotopic liver transplants due to complications of hepatic sarcoidosis. Kennedy et al. [41] approximately one-third of the patients had a complete clinical response, one-third had a partial response, and one-third showed no response. Graf et al. [40] found that 69.4% of patients were treated with glucocorticoids and 40.3% with ursodeoxycholic acid (UDCA). ALP levels decreased by 60.8% with glucocorticoids and 59.9% with UDCA. Few patients needed a second-line agent, with eight achieving normalized ALP levels during follow-up. These studies show that corticosteroids, antimetabolites, and immunosuppressants are important in controlling disease. Hepatic sarcoidosis is a rare indication for orthotopic liver transplantation. There are only a few case reports and small series evaluating the outcomes of these recipients. In our review, we found that liver transplantation provides satisfactory long-term patient and graft survival for patients with liver failure due to hepatic sarcoidosis. The incidence of disease recurrence in the liver is not known. However, it appears to have a minimal impact on the long-term outcomes for both patients and grafts [45,46,47,48,49,50,51,52,53,54,55,56,57,58,59,60,61,62,63,64,65,66,67,68,69,70,71,72,73,74,75].

### 3.4. Splenic Sarcoidosis

Splenic sarcoidosis is characterized by granulomatous involvement of the spleen, observed in 40–80% of systemic sarcoidosis cases, although symptomatic manifestation is infrequent, occurring in less than 5% of cases [76,77,78,79,80,81,82]. Symptomatic presentations include splenomegaly, which may be associated with cytopenia (such as anemia, leukopenia, thrombocytopenia, or pancytopenia), spleen infarction, and abdominal pain localized to the left upper quadrant. The diagnosis is based on clinical evaluation and radiologic imaging, typically ultrasound or computed tomography (CT) scans. Therapeutic intervention with corticosteroids is indicated primarily in instances of hypersplenism or significant splenomegaly. In rare cases, where there is an insufficient response to pharmacological management or the presence of severe complications, splenectomy may be warranted [82,83,84,85].

### 3.5. Pancreatic Sarcoidosis

Pancreatic involvement in systemic sarcoidosis is rare, with autopsy studies showing a prevalence of 1–5% and even lower rates in clinical series. Pancreatic sarcoidosis can present itself with nodular abnormalities or as a mass [46,85,86,87].

Since their initial description in 1950 in the study of Curran et al. [88], only 25 cases of symptomatic sarcoidosis presenting as a pancreatic mass have been documented. Symptoms of pancreatic sarcoidosis, resulting from tissue infiltration or bile duct obstruction, often resemble those of pancreatitis or pancreatic cancer. Non-surgical biopsy methods, such as CT or endoscopic ultrasound, have proven unreliable for diagnosing pancreatic sarcoidosis. A definitive preoperative diagnosis of sarcoidosis with biopsy is crucial for avoiding unnecessary pancreatic surgery [85,86,87,88,89,90,91].

### 3.6. Large Intestinal Sarcoidosis

Large intestinal sarcoidosis is often nonspecific in its presentation, with weight loss, diarrhea, constipation, and abdominal pain being the most encountered symptoms [92]. In one case, a patient’s large intestinal sarcoidosis presented as an endoluminal mass. Diagnosis, as per the ACCESS system, requires a compatible biopsy presenting non-caseating granulomas and exclusion of any other similar possible causes of the disease [93]. In five of the articles, sarcoidosis was either considered to be a tumor initially or was concomitant to one, usually large intestine adenocarcinoma. A possible novel approach to differential diagnosis between adenocarcinoma of the large intestine and sarcoidosis might lie in a comparison between Fluorodeoxyglucose-18 (FDG-18) and ^68^Galluim-citrate Positron Emission Tomography-Computerized Tomography (PET-TC), seeing how the two entities present discrepancies when examining uptake rate, with sarcoidosis resulting positive exclusively in the FDG-18 scan. In several cases, the localization of the disease activity was not limited to the large intestine, which existed in other districts as well, with cutaneous sarcoidosis being the most frequently associated [94,95].

Large intestine involvement in sarcoidosis remains an extremely rare occurrence, and as such, it is difficult to estimate its prevalence within society and the population of patients affected by it. Treatment of large intestinal sarcoidosis remains like that of most other organs involved, with high-dose oral steroids being the first line of treatment, methotrexate second, and finally, monoclonal antibodies such as adalimumab third [9]. The initial approach is conservative, with surgical intervention being reserved for refractory cases, like the indication for organ transplant in pulmonary sarcoidosis in the most recent ERS and ATS guidelines [96]. In certain instances, dedicated, supportive treatment is required, such as in cases of excessive bleeding and consequent anemia, where blood transfusions may be considered [97].

## 4. Discussion

GI involvement is rare, typically asymptomatic, and reported in only 0.1–0.9% of sarcoidosis cases. When present, GI sarcoidosis can significantly impact patient health, particularly due to diagnostic challenges. Reflux is a frequent manifestation, often accompanied by symptoms such as epigastric pain, nausea, vomiting, and weight loss. The stomach is the most affected GI organ, noted in approximately 10% of cases. Gastric sarcoidosis primarily presents as either gastric ulcer formation due to localized mucosal infiltration or diffuse granulomatous infiltration leading to reduced lumen size secondary to fibrosis [3,98,99].

Diagnosis is complicated by the similarity of GI sarcoidosis symptoms to other GI conditions, including irritable bowel syndrome (IBS), GERD, gastroparesis, and gastric cancer. This reinforces the need to consider an accurate differential diagnosis to establish appropriate treatment [10,50,98,99,100]. The prognosis for patients with gastric sarcoidosis varies, with complications such as strictures, fistulas, or chronic malabsorption potentially occurring, especially if the disease is not adequately controlled. Additionally, cases have been reported in which patients with gastric sarcoid developed mucosa-associated lymphoid tissue lymphoma, hematological malignancies, and gastric adenocarcinoma, all of which negatively impact the patient’s prognosis [3].

Hepatic sarcoidosis, a more common extrathoracic manifestation, is often asymptomatic but can present with signs of cholestasis. Due to the lack of specific serological markers, diagnosis relies upon a high index of clinical suspicion, physical examination, laboratory abnormalities, and histological confirmation. In rare cases involving the small bowel and large intestine, histological diagnosis is essential to exclude more common diseases. Increased clinical awareness and suspicion of hepatic sarcoidosis are crucial for the timely diagnosis and introduction of effective treatment [17,40,41,42,43,94,101,102,103].

The monitoring of GI sarcoidosis is achieved clinically and radiographically, but there is no evidence of the role of serum angiotensin-converting enzymes or serum interleukin-2 receptors in monitoring. Asymptomatic sarcoidosis does not require treatment. For symptomatic patients, glucocorticoids (GCs) can reduce liver and spleen size, decrease granulomas, and improve organ function to some extent. However, their effect on disease progression and complications like portal hypertension or hepatic fibrosis is limited. Steroid-sparing agents, defined previously as ursodeoxycholic acid, have shown some success. Ursodeoxycholic acid is particularly used for cholestatic jaundice. Despite preferences for AZA over MTX due to hepatotoxicity concerns, both drugs pose known risks [44,45,46,47,48,49,50,51,104,105]. In end-stage liver disease, orthotopic liver transplantation has been successfully reported, with recurrence rates in the allograft comparable to other diseases [43,67,68,69,70,71,72,73]. Splenectomy is considered only in rare cases of huge splenomegaly, severe hypersplenism, suspected malignancy, or to prevent splenic rupture when medical treatment fails.

Today, developing new therapeutic and diagnostic protocols for GI sarcoidosis is necessary to improve patient outcomes and address the complexities of this rare but impactful disease manifestation.

## 5. Conclusions

Patients with known systemic sarcoidosis, particularly those with extrapulmonary manifestations, are more likely to develop GI involvement. Despite this, GI involvement in sarcoidosis remains an under-recognized complication with significant implications for patient morbidity and mortality. To date, there is a paucity of dedicated clinical trials that establish the efficacy of anti-inflammatory agents in this context. Future research endeavors must develop diagnostic (including novel biomarkers) and therapeutic protocols aimed at early detection and managing the disease burden and activity, thereby mitigating its progression.

## Figures and Tables

**Table 1:** Most common symptoms associated with gastric sarcoidosis.

Symptom	Incidence	Mention
Weight Loss	30%	Afshar K et al. [3]
Dyspepsia	20%	Espinel J et al. [29]
Upper Gastrointestinal Bleeding	10%	Afshar K et al. [3] Leeds JS et al. [30]
Early Satiety	15%	Leeds JS et al. [30]
Bloating	10%	Kariyanna PT et al. [31]
Anemia	5%	Kariyanna PT et al. [31]

**Table 2:** Studies showing the major outcomes for hepatic sarcoidosis after drug therapy and number of liver transplantation.

Reference	Population	Drug Therapy	Outcomes	Liver Transplant
Graf et al. [40]	n = 62 (f = 51.6%, m = 80.6%)	Glucocorticoids (n = 43) Ursodeoxycholic acid (n = 25) MTX (n = 9) MMF (n = 1) CMF (n = 2) Infliximab (n = 1)	Patients who needed a second-line immunosuppressive (n = 17) Deaths for liver-related complications (n = 3)	n = 0
Kennedy et al. [41]	n = 180 (f = 89, m = 91)	Glucocorticoids, MTX, MMF or CMF Infliximab	Patients who received a second-line immunosuppressive (n = 16)	n = 6
Sedki et al. [42]	n = 286 (f = 223, m = 63	Glucocorticoids (n = 17) MTX, MMF or CMF (n = 15) Infliximab (n = 5)	Patients who responded well to treatment with normalization of liver biochemistries (n = 18) Deaths for liver-related complications (n = 0)	n = 2

f = females. m = males, MMF = mycophenolate, CMF = cyclophosphamide, MTX = methotrexate.

## Appendix A

**Table A1:** Case reports describing symptoms, treatment, and outcomes of gastric sarcoidosis.

Case	Symptoms	Diagnosis	Treatment	Outcomes
El Sharu H. et al. [106]	Abdominal pain, nausea, vomiting, diarrhea	CT of abdomen and pelvis, MR angiography, EUS, chest CT, head CT, head MRI	Prednisone 40 mg	Symptomatic improvement, persistence of symptoms following prednisone tapering
Tao J et al. [107]	Chest pain, shortness of breath, abdominal cramping	Chest CT, PET, left thoracotomy, EGD, colonoscopy	Prednisone	Complete resolution of symptoms in five months
Hassan D. et al. [23]	Abdominal pain, nausea, vomiting, weight loss	CT of abdomen and pelvis, barium swallow series, EGDS	Adalimumab and methotrexate	Continued hospital and clinic presentations with nausea, vomiting, and abdominal pain
Basida B et al. [108]	Abdominal pain	Chest X-ray, CT abdomen, CT-guided pigtail catheter placement, EGD, colonoscopy	Followed by outpatient lung biopsy	-
Gala K et al. [32]	Dyspepsia, nausea	EGDS	Pantoprazole 40 mg	Slight improvement
Sameh S et al. [26]	Abdominal pain	EGDS, chest CT, bronchoscopy	Corticosteroids	Favorable clinical course
Mota C et al. [109]	Abdominal pain, vomiting, abdominal distension, asthenia, anorexia	Body CT scan, EGD, colonoscopy, bronchoscopy, laparoscopy	Noteworthy corticosteroid therapy in intensive care unit	Regression of lesions, pleural effusion, ascites, no distension of intestinal loops
Deepa A et al. [110]	Dysphagia, nausea, vomiting	CT abdomen, CT chest, EGDS, bronchoscopy	Steroids for about 6 months	Regression of lymph nodes and decreased GI symptoms, relapse after 5 months of treatment interruption
Stemboroski L et al. [27]	Chronic diarrhea and abdominal pain	CT abdomen and pelvis, EGDS, colonoscopy	Prednisone 40 mg daily	Resolution of diarrhea and abdominal pain within 3 weeks
Kariyanna PT et al. [31]	Epigastric pain, dysphagia, vomiting, weight loss	Chest X-ray, EGDS	Prednisone 50 mg daily, omeprazole 20 mg, cosyntropin 80 UI (once every 3 days)	Improvement of symptoms
Ceylan E et al. [25]	Chest tightness, inability to take a deep breath	Chest and abdomen CT, PET/CT, EGDS	Partial pleural decortication, wedge resection for lung nodule, pantoprazole 40 mg, metoclopramide hydrochloride 10 mg, sucralfate 2 g	Occasional GI symptoms in 1 year of FU. Persistence of non-caseating epithelial antrum granulomas with normal EGDS
Tokala H et al. [111]	Epigastric pain, nausea, vomiting, weight loss	CT abdomen and pelvis, EGDS	Prednisone 60 mg	Alleviation of symptoms within four days, no recurrence of symptoms in 2 years after corticosteroid tapering
Shkolnik LE et al. [112]	Abdominal pain, vomiting	Chest radiograph, CT abdomen and pelvis, hepatobiliary scan, abdomen MRI, EGDS	6-month course of systemic corticosteroid	Complete resolution of symptoms
Case 1. (Inomata M et al.) [113]	Epigastric discomfort, weight loss	Chest X-ray, CT, MRCP, EGDS, liver biopsy, barium scintigraphy	Prednisolone 30 mg	Improved symptoms and laboratory values, died 11 months later from MOF and DIC due to infected AAA
Case 2. (Inomata M et al.) [113]	None	Chest X-ray, CT, EGDS, barium scintigraphy	Conservative	No symptoms for 5 years after, follow-up EGDS 1 year later showed no remarkable changes
Case 3. (Inomata M et al.) [113]	-	Chest X-ray, CT scan, EGDS, barium scintigraphy	PPIs	Slight epigastralgia continued for two and a half years, follow-up EGDS 1 year later revealed a scar on the greater curvature
Chaudhary P et al. [114]	Generalized fatigue, early satiety, weight loss	Bone marrow biopsy, CT abdomen, EGDS	Prednisone, B12, and iron supplementation	Gained weight, normalization of neutrophil and white blood cell count

**Table A2:** Case reports describing symptoms, treatment, and outcomes for small bowel sarcoidosis.

Case	Other Disorders	Symptoms	Diagnosis	Treatment	Outcome
Paone G et al. [39]	-	Nausea, vomiting, abdominal pain, constipation, fever	Abdominal X-ray, CT of abdomen and pelvis, Total body CT	Laparotomy, Prednisone 25 mg/daily (slowly tapered)	Complete response to therapy in 3 months
Pendela VS et al. [38]	Kidney stones	Acute right iliac fossa pain, vomiting, chronic low back pain	Abdominal ultrasound, CT scan of the abdomen, MRI of the spine, 18 FDG PET, Bronchoscopic ultrasound-guided biopsy	Laparoscopic appendectomy: steroid 30 mg daily (slowly tapered), added mycophenolate mofetil after six months	Pain resolved postoperatively, and decrease in the size of the vertebral lesions
Zakaria A et al. [37]	Inactive pulmonary sarcoidosis	Nausea, vomiting, abdominal pain, constipation	Abdominal X-ray, CT scan of the abdomen and pelvis, Gastrografin small-bowel follow-through, Diagnostic laparoscopy	Conservative treatment (bowel rest, nasogastric tube, IV fluids) followed by prednisone taper dose	Good response after corticosteroid therapy
Esmadi M et al. [36]	Diabetes, previous Potus, and mediastinal lymphadenopathy, Liver biopsy	Abdominal pain, severe peripheral arthralgias, chronic diarrhea	X-ray and CT abdomen and pelvis, Abdominal ultrasound, Bone marrow biopsy, Colonoscopy	Prednisone	Improvement in his symptoms
